# Ten Simple Rules for Starting a Company

**DOI:** 10.1371/journal.pcbi.1002439

**Published:** 2012-03-29

**Authors:** Anthony C. Fletcher, Philip E. Bourne

**Affiliations:** 1 Salix Management Consultants Ltd, London, United Kingdom; 2 Department of Pharmacology, University of California San Diego, La Jolla, California, United States of America; 3 Skaggs School of Pharmacy and Pharmaceutical Sciences, University of California San Diego, La Jolla, California, United States of America

Many faculty, staff, and students at academic institutions think about starting companies at some point in their careers. As academic funding models change, and how academia views entrepreneurial activity changes, starting companies is likely to happen more frequently. Hence, it is worth considering *Ten Simple Rules* to contemplate when starting a company while in academia. There is a wealth of general information out there to help you, but that information is not aimed specifically towards computational biologists. What follows is a hybrid that is intended as a general quick review for anyone, intermingled with some specific advice for computational biologists.

By way of experience, we should say we have been involved in starting several companies: both in the biomedical sciences, dealing with biological software, computational biology services, and currently SciVee Inc. (http://www.scivee.tv) distributing scientific rich media, and outside it, ranging from the distribution of independent films, to a socially oriented dining club aimed at supporting local businesses, to a business supplying art quality photographic prints. None have been a great financial success, but all have been immense fun, an opportunity to meet interesting people, and an opportunity to think quite differently than when doing scientific research. Read that as a personal endorsement to go for it, even if starting a company is not yet a well-formed idea, and even though, as you will see below, the rules themselves might be cautionary and off-putting.

## Rule 1: A Great Product or Service Alone Is Not Enough to Make a Successful Business

This is a general rule of business. Very rarely is a product alone unique and desirable enough to make a successful business, even though the majority of academic founders tend to think so at the beginning. To misquote Ralph Waldo Emerson, “Make a better mousetrap and the world will beat a path to your door” is perhaps one of the most misleading axioms ever circulated. It is useful to continuously remind oneself that at least nine out of ten companies fail: indeed, most VCs (venture capitalists) will tell you that most business ideas they see should be canned before they ever reach the business plan stage, let alone before serious money is spent on development. Success comes from a huge amount of hard work, extensive market research, a realistic business model, and, above all, the application of good all-around business skills to all aspects of the business. For example, if you are great at sales but poor at money management, your business will certainly fail. Notwithstanding, in one sense the process of starting a business is not that different from academic research (replace business plan by grant and think about current grant funding levels), but it is the way the process is executed that is alien to most academics. Hence Rule 2.

## Rule 2: Business Is Part Art, Part Science

Success as an academic comes from having an eye for detail and contemplating a problem from every conceivable angle and only moving forward when you believe you have fully analyzed the problem. Businesses are typically different—the ecosystems involved are very complex, so they cannot be tested or researched to anything like the same degree of certainty. On occasion you must act quickly and based only on gut instinct. It is not by chance that economics is referred to as the “dismal science”. There is a degree of luck behind every success.

## Rule 3: Define to Yourself and Others Why You Are Starting a Company and Be Prepared for Those Reasons to Change

There are many reasons academics start companies. At one end of the spectrum is the desire to make money; at the other end is the desire to do something for humanity. For most of us the reason is usually somewhere in between. That sounds fine, but in practice the time will come when one has to make hard choices and sacrifice one for the other. At the outset, define for yourself and others who are working on establishing the company where each of you stands on this issue, what is motivating you, and what you are willing to sacrifice. Remember also that a company is a legal entity in its own right and exists only to serve its stakeholders and no one else. As a rule of thumb, it often takes three CEOs to take a company to floatation—one to start it, one to develop it into a thriving business, and one to float or sell it—all different skills. Boards and executives will come and go and companies will change over time as they have to adapt to a market and not the other way round. Consequently, once you have breathed life into a company it can continue, with or without you, and its shape and purpose may change radically as markets demand.

## Rule 4: Decide What of Yourself You Are Willing to Put into the Company

There are many forms of investment to start companies—ranging from your own credit card or bank loan, angels (private investors), institutions, or VCs. It is a rare business that can be started on its own internal cash flow or is unique in its offerings to the marketplace. A common metric used by investors as part of evaluating a company is to ask the founders what money and time they are willing to invest in the company. The answer is telling to them and to you. Most academics will not give up their day jobs to establish a company, which is a red flag for investors unless you can immediately compensate for this issue with the required resources, such as an experienced CEO or other team members. Hence Rule 5.

## Rule 5: Get Professional Business Help Early

Starting a company will require many skills an academic does not typically have. The general outline of a business plan describes these amongst others:

- Product/manufacturing/service delivery
- Manufacturing process
- Sales and marketing
- Business management and administration
- IT
- HR/personnel
- Financial management
- Legal, patents, contracts, etc.

The needed depth of such skills will also vary depending on the maturity of the business.

You can learn some of these skills as you go and there is fun in that, but for some tasks you will undoubtedly need deep expertise that can only be had from business professionals: for example, don't write your own contracts—get a lawyer to review them and don't expect to go from your laboratory and become a competent and successful salesperson. Above all, get a decent bookkeeper or accountant on board, as nothing is more depressing than spending your evenings doing the accounts and invoicing! So, recognize that you will need help and that help has to be paid for in some way—either through equity or more commonly, actual money—your shares in a start-up don't pay the weekly bill at the supermarket! There are consultants and professional firms that specialize in helping start-ups, and of course most are upright and professional. But not everyone is, so get some advice from business professionals you trust before settling on a course of action that may be irreversible.

## Rule 6: Understand the Legal, Ethical, and Regulatory Environment Thoroughly

Companies operate in bureaucratic and litigious environments unfamiliar to most academics. The key here is to get the right advice early on—you won't be able to proceed without lawyers, accountant, insurers, and many other professionals. But, remember their interests and yours are not necessarily aligned even though you are their client. So the rule here is to do your own research first and understand what is a “must have” and what is simply an “optional extra”. You can then operate in a pragmatic way and keep your expenditure of time and resources to a reasonable minimum. Finally, remember that investors will expect you to have covered the bases properly and be operating on advice from recognized experts, which is usually expensive.

## Rule 7: Establish a Relationship with Your Academic Institution at the Outset

You need to understand the relationship you as an institutional employee and the company you are founding have with the academic institution in which you work. Typically, if the product comes from your laboratory, the institution in which you work owns the intellectual property (IP) and the company needs to license it from the institution. The work that produced the IP, and perhaps the associated product(s), was likely funded by a third party, and their relationship to the institution and to the company needs to be established. Typically, a government funding agency will confer ownership on the institution in which the work was done. Where things get tricky is when the IP and/or associated product(s), or follow-on IP and product(s), are developed in the company, what is your institution's rightful share? This is where the lawyers or other negotiators come in to establish IP rights.

## Rule 8: Realistically Define the Value of Your Business

It is easy to believe that the initial value of your product, and hence company, is much greater than it is. We all read of those rare cases where a start-up is sold to Google or Microsoft for a small fortune, but they are the exceptions. For example, in computational biology, a company based solely on software has little to no value. Your heart and soul may have gone into developing the software, but the hard fact is that others can likely recreate it, and it is difficult to protect; therefore, to an investor it has little value. What does have value is your experience in using that software to achieve the desired outcome, which is a service business with typically a limited but targeted audience. On top of that, the software must be of commercial quality that requires quality assurance, professional technical writers, a support/help desk, etc. Professional marketers call this the unique selling proposition—it's what separates you from your competitors and why people will buy from you—sometimes it's the product, sometimes the service, and sometimes the people, or a combination of all things. Whatever it is, it must be clear and compelling, and then it's a straightforward matter to put some metrics around it, work out what it's worth, and see who is interested.

## Rule 9: Think about Conflict of Interest Every Day

This is a serious issue that could impact your standing in the university and your community of scientific peers. Your professional standing will likely always be more valuable to you than being a co-founder of a company, so be careful. Many institutions have a conflict of interest office that can assist you in making the right decisions. The bottom line is that nothing you do in the laboratory should be driven by what can be perceived as being for the sole benefit of the company. Obviously, there is a huge gray area when it benefits the laboratory and likely the company as well. A useful exercise before undertaking something that might be perceived as a conflict is to conjure up how someone who didn't like you could spin what you are doing as a negative article in a reputable newspaper. How does that article read? Academic institutions competing for students and research funds do not like bad publicity.

## Rule 10: Decide Responsibilities and Equity Share before You Start

The nicest people and the best collegial relationships will be tested when forming a company together. The farther you go in establishing the company, the more vested each of those involved will become in its success. This is good on the one hand, but very bad if expectations for reward are not set at the outset. When incorporating the company, an initial set of shares is assigned; this is the time to define the initial equity share and should be based on past contributions to the enterprise and what each founder and employee will contribute to the company. Shares that vest over time (i.e., potential value that increases the more time and effort you put into the company) are a typical means of defining on-going contributions as well as monetary rewards. We would go so far as to say that in the life of a company there are always falling outs: the advantage of a limited company is that company rules typically state what should happen in such circumstances and the shareholders decide collectively. So never go ahead without planning for some acrimony and never ever go ahead on the basis of a handshake or as an unwritten partnership—lawyers are expensive in resolving conflicts.

As we said at the outset, the rules are a cautionary tale, but do not be deterred. Businesses are hard work but they are fun; you'll enjoy the experience. Nothing really beats the handshake (and signing) that closes a sale, or the unsolicited testimonial that tells you what you produced helped someone. You'll meet lots of people you would never have met before and probably go to places (nice and not so nice) that you would not go as an academic. You will take pride in having created something tangible that people value; they paid money for it after all. But remember never to compromise your academic principles—they are the most valuable asset you have.

**About the Authors**

**Anthony C. Fletcher** has a PhD in organic chemistry, and had a 20-year career as a management consultant with Deloitte and with Cap Gemini Ernst and Young, and is now a freelance consultant.

**Philip E. Bourne** was trained as a physical chemist, is a professor of Pharmacology at the University of California San Diego, and the co-founder of SciVee.tv and editor-in-chief of this journal. The authors have known each other since birth, still go motorcycling together, and have been involved in two companies together.

